# Primary care obesity management in Hungary: evaluation of the knowledge, practice and attitudes of family physicians

**DOI:** 10.1186/1471-2296-14-156

**Published:** 2013-10-19

**Authors:** Imre Rurik, Péter Torzsa, István Ilyés, Endre Szigethy, Eszter Halmy, Gabriella Iski, László Róbert Kolozsvári, Lajos Mester, Csaba Móczár, József Rinfel, Lajos Nagy, László Kalabay

**Affiliations:** 1 Department of Family and Occupational Medicine, Faculty of Public Health, Medical and Health Science Center, University of Debrecen, Nagyerdei krt. 98, 4032, Debrecen, Hungary; 2 Department of Family Medicine, Faculty of Medicine, Semmelweis University, Budapest, Hungary; 3 Hungarian Society for the Study of Obesity, Budapest, Hungary; 4 Institute of Family Medicine, Faculty of Medicine, University of Szeged, Szeged, Hungary; 5 Irinyi Primary Health Care Center, Kecskemét, Hungary; 6 Institute of Family Medicine, Faculty of Medicine, University of Pécs, Pécs, Hungary

**Keywords:** Attitudes, Family physician, General practitioner, Hungarian, Knowledge, Management, Obesity, Overweight, Practice, Survey

## Abstract

**Background:**

Obesity, a threatening pandemic, has an important public health implication. Before proper medication is available, primary care providers will have a distinguished role in prevention and management. Their performance may be influenced by many factors but their personal motivation is still an under-researched area.

**Methods:**

The knowledge, attitudes and practice were reviewed in this questionnaire study involving a representative sample of 10% of all Hungarian family physicians. In different settings, 521 practitioners (448 GPs and 73 residents/vocational trainees) were questioned using a validated questionnaire.

**Results:**

The knowledge about multimorbidity, a main consequence of obesity was balanced.

Only 51% of the GPs were aware of the diagnostic threshold for obesity; awareness being higher in cities (60%) and the highest among residents (90%). They also considered obesity an illness rather than an aesthetic issue.

There were wider differences regarding attitudes and practice, influenced by the the doctors’ age, gender, known BMI, previous qualification, less by working location.

GPs with qualification in family medicine alone considered obesity management as higher professional satisfaction, compared to physicians who had previously other board qualification (77% vs 68%). They measured their patients’ waist circumference and waist/hip ratio (72% vs 62%) more frequently, provided the obese with dietary advice more often, while this service was less frequent among capital-based doctors who accepted the self-reported body weight dates by patients more commonly. Similar reduced activity and weight-measurement in outdoor clothing were more typical among older doctors.

Diagnosis based on BMI alone was the highest in cities (85%). Consultations were significantly shorter in practices with a higher number of enrolled patients and were longer by female providers who consulted longer with patients about the suspected causes of developing obesity (65% vs 44%) and offered dietary records for patients significantly more frequently (65% vs 52%). Most of the younger doctors agreed that obesity management was a primary care issue.

Doctors in the normal BMI range were unanimous that they should be a model for their patients (94% vs 81%).

**Conclusion:**

More education of primary care physicians, available practical guidelines and higher community involvement are needed to improve the obesity management in Hungary.

## Background

In Hungary, as in many other countries, overweight and obesity are becoming an epidemic and they are responsible for most of the pathologic conditions [1,2]. Obesity epidemic is a challenge to public health and requires medical interventions, individual behavior modifications and environmental changes [3]. Obesity is an epidemic among primary care patients as well. While family physicians care for the consequences of obesity, they do not generally feel confident about managing obesity itself [4]. The majority of medical consultations take place in primary care settings and general practitioners (GP) have an opportunity to observe their obese patients’ weight gain for decades [5,6]. The physician’s knowledge is a basic tool which should be permanently improved. The doctor’s daily practice in this area should be based on guidelines and recommendations; plenty are available about the complications of obesity such as diabetes, cardiovascular diseases and risks and their management.

High prevalence of negative attitudes was found, particularly among younger physicians and those with lower patient volume. Broader knowledge of weight-loss diets was associated with less dislike in discussing weight loss, less frustration, greater trust in the efficacy of treatment, and less pessimism about patient success [7]. Knowledge gaps and ambivalent attitudes toward obesity management were found among GPs in different countries. In addition, frustration with the resources and structure of current primary care systems, overburdening of outpatients consultation prevented them from dealing with obesity in the proper way [4,8,9]. Many studies noted that physician’s recommended healthy lifestyle (increased physical activity), dietary advice (decreased number of total calories) or referral to a dietician but rarely provided a practical programme of how to implement these recommendations. It is obvious that there is a need for education of primary care physicians to increase the uniformity of the assessment and improve physicians’ self-efficacy in managing adult and childhood obesity [8,10]. Physicians often report a lack of confidence in managing obesity. Lack of patient motivation is perceived to be the greatest barrier. Physicians with greater knowledge, more positive attitudes toward obesity management, and access to more resources are more likely to provide weight management in primary care settings [11].

A systematic review has found that obesity is a stigmatized condition that exerts a negative impact on the relationship between patients and health-care providers. The presence of obesity affects health-care interactions and decision making [12].

### Aim

The aim and research question of this study is to assess Hungarian general practitioners’ knowledge, attitudes, practices, their interactions and find barriers with other factors that influence the physicians’ willingness and ability to manage obesity.

## Methods

### Study design

#### Cross sectional survey

An anonymous questionnaire based on a validated internationally published questionnaire was developed and validated again in own language by the primary care experts of all Hungarian medical faculties [13].

Data were asked about the doctors’ gender, age, working domicile, board specifications, and practice characteristics, demography, number of enrolled patients. There were questions focussing on numerical data to explore how practitioners estimated the ratio of obese or overweight patients in their practice.

Altogether 81 (mostly multiple choice) questions were asked in three main domains (knowledge, attitude and professional practice). The results were presented in the same way.

### Settings

Different educational events of family physicians and participants of a residency programme in family medicine, organized by the four departments of family medicine in Hungary in 2011, where the printed version of the questionnaire was distributed. Altogether 523 questionnaire for GPs and 78 for residents were delivered but only 448 and 73 was completed, ready for data recording. It means a response rate of 86% and 92% respectively.

### Selection of participants

Participation was voluntary without any financial incentives.

### Exclusion criteria

Refusal of participation for any reason or partially completed questionnaire.

### Data sources

Completed questionnaire from GPs and residents.

### Quantitative variables

Derived from the answers given to the questionnaire.

### Qualitative variables

Outcome of *factorial* analysis, describing the characteristics of participants.

### Ethics

According to the recent Hungarian regulations, surveys among health professionals do not require previous ethical permission [14].

### Statistics

*ANOVA, unpaired* and *paired t-, Fisher’s exact- and chi-square tests* were used in order to explore connections between the answers and the main characteristics of the study population. P < 0.05 was considered statistically significant.

For more sophisticated comparison factorial analysis using *Kaiser-Meyer-Olkin* measure was also performed to describe the respondents’ characteristics. Using a *dendogram*, derived from the *Ward hierarchic way* three clusters were established based on the questions relating to the following qualitative characteristics:

–Sense of vocation in the treatment of obesity;

–Professional skills in managing obesity;

–Counseling for obese patients.

All of the analyses were performed using STATA 10.1.software (Statacorp LP. College Station, TX, USA).

## Results

### Descriptive data

Altogether 448 family physicians (170 male and 278 female) and 73 residents (18 male and 55 female) completed the questionnaire. The GPs’ mean age was 54.5 ± 9.8 years, the youngest and the oldest respondents being 31 and 82 years old, respectively. The residents’ mean age was 29.9 ± 5.4 years.

The average number of enrolled patients in the practices was 1675 ± 483. There were 308 practices with adults population and 56 family pediatricians (having children population only (<14 years). In 84 practices, all generations were under care. The geographical locations of practices were as follows: 119 in Budapest, 99 in big, 126 in smaller cities and 104 in villages.

The GPs spent 19.3 ± 11.2 years on average in the practice and the mean of board-qualification was 1.9 ± 0.9. Of them 150 were specialized in family medicine only, 199 had two, 70 had three and 29 had more board qualifications. It means that they started their carrier in other professional fields, mainly internal medicine, and later on they changed their job/position in family medicine and become qualified in this field as well. The recent anthropometric characteristics of doctors are given in Table 1.

**Table 1:** The mean of BMI, BMI categories and number of responders

	**GPs**			**Residents**		
	**male**	**female**	**total**	**male**	**female**	**total**
BMI [kg/m^2^]	27.2	25.0	25.8	26.8	21.7	
*±SD*	*4.9*	*4.6*	*4.7*	*3.7*	*3.5*	
BMI categories [kg/m^2^]						
Underweight (<18.5)	2	5	7		7	7
Normal (18.5-24.9)	56	158	214	7	41	48
Overweight (25–29.9)	72	78	150	7	6	13
Obese (30 < )	40	37	77	4	1	5
total	163	274	448	18	55	73

### Outcome data

In their own practices, the incidence of overweight among adults was estimated as 34.3%, that of obesity as 23.4%, with small differences between different types of settlements. It was almost the same among children.

Answers and their relation to the descriptive data are presented in 3 domains.

#### Knowledge

Questions focusing to *causes and consequences of obesity* tried to explore the GPs’ knowledge. There were also statements and responders were requested to agree or disagree with them (Table 2). Hormonal, genetic and environmental factors were rated among the main causes of obesity, mainly long term positive energy balance, combined with physical inactivity, besides impaired psychological conditions. There was a wide consensus about consequences and statements as well.

**Table 2:** Knowledge on causes, consequences an statements of obesity and distribution of answers in this domain

**Knowledge**	**Disagree**	**Agree**
**Causes of obesity**	**[%]**	**[%]**
Eat too much fat	11.0	89.0
Insufficient physical activity	2.8	97.2
Genetic factors	26.8	73.2
Repeated dieting	41.6	58.4
Stress, anxiety and depression	21.5	88.5
Hormonal disorders	22.9	77.1
Low income, unemployment	54.3	45.7
**Consequences of obesity**		
Medical problems	4.9	95.1
Psychological problems	5.5	94.5
Social problems	15.8	84.2
**Statements**		
Obesity is a disease	11.5	88.5
Normal weight is important in health	4.4	95.9
For overweight and obese patients even small weight loss can produce health benefits	5.2	94.8
Most **overweight** patients should be treated for weight loss	9.9	90.1
**Only obese** patients should be treated for weight loss	70.0	30.0

The BMI thresholds for diagnosing overweight / obesity were defined exactly only by 51.3% of doctors. Physicians working in the capital (37%) and villages (47%) were significantly less prepared, than their colleagues in the cities (≈60%). Residents who participated in vocational training were better educated; more than 90% of them had a reliable knowledge on diagnostic thresholds. Younger doctors were more unanimous in considering obesity as an illness compared to those who believed it was a symptom only (53.9 ± 0.5y vs 57.8 ±1.5y, p = 0.01).

### Statement

Seventy percent of the physicians disagreed that body weight should be decreased in obese patients only. This ratio was 55.1% among pediatricians.

This approach was influenced by the doctors’ own BMI. Although many agreed that weight reduction was expected not only from obese patients, only 46.7% of doctors with BMI over 30 kg/m^2^ had the same opinion (p = 0.007). The mean age of those who agreed was significantly higher (56.2 ± 0.9 years vs 53.7 ± 0.6 years, p = 0.019).

#### Attitude

The GPs’ different personal attitudes, bias and preconceptions were explored by using the questionnaire. Willingness and self-confidence were quite different as well (Table 3).

**Table 3:** GPs’s attitudes concerning obesity and obese persons and distribution of answers in this domain

**Attitudes**	**Disagree**	**Agree**
	**[%]**	**[%]**
GP’s role is to refer overweight or obese patients to other professionals rather than attempt to treat themselves	83.7	16.3
GPs should be models and maintain normal weight	11.2	88.8
I feel well-prepared to manage overweight and obese patients	43.4	56.6
Treating overweight and obese patients is professionally gratifying	29.5	70.5
Obese patients are lazier and more self-indulgent than people with normal weight	32.1	67.9
Overweight patients are lazier and more self-indulgent than people with normal weight	34.4	65.6
Only a small percentage of overweight and obese people can lose weight and maintain this loss	20.4	79.6

The mean age of those who believed obesity could be managed in primary care without sending all obese patients to specialists was lower (53.7 ± 0.5 years vs 58.4 ± 1.1 years, p = 0.04).

Ninety-four percent of doctors in the normal BMI category agreed that family physicians should be an example in body weight, while only 80.8% of obese doctors gave the same answer (p = 0.004).

Treating obesity means a higher professional satisfaction for doctors having board qualification in family medicine only compared to others who are more qualified (76.8% vs 67.6%, p = 0.038).

The belief that “*Obese* patients are lazier and more self-indulgent than people with normal weight” was supported by 52.6% of obese and 67.9% of *non-obese* doctors (p = 0.01). The mean BMI of those doctors who agreed was 25.5 ± 0.21 kg/m^2^, while it was higher (26.5 ± 0.4 kg/m^2^) among those who disagreed (p = 0.026). This belief regarding patients in the *overweight* category was supported by 54.8% of obese doctors and 65.8% of those who were categorized with lower BMI (p = 0.036). Doctors who agreed with this statement were older (55.1 ± 0.5 years) than those who disagreed (52.7 ± 0.8 years, p = 0.024).

#### Practice

The diagnostic, treatment and consultation practices were not uniform. Anthropometric parameters related to obesity as diagnostic tools were considered differently (Table 4).

**Table 4:** Diagnostic practice, advice given and tools used by GPs in the management of obesity with the distribution of answers in this domain

**Practices**	**Disagree**	**Agree**
**Diagnostic methods**	**[%]**	**[%]**
Weight without reference to height	54.9	45.1
BMI calculation	20.9	79.1
Waist / hip ratio	44.4	55.6
Waist measurements	34.9	65.1
Comparison with ideal weight	30.2	69.8
Appearance	77.9	22.1
**Weight management advice and tools**		
Eat less during meals	12.9	87.1
Eat less fat	10.2	89.8
Don’t eat between meals	23.3	76.7
Eat less sugar	6.7	93.3
Eat more fruits and vegetables	3.7	96.3
Consume fewer caloric drinks	5.3	94.7
Definitely avoid specific foods	31.0	69.0
Follow personalized **low-calorie** diet (1200–2200 kcal/day)	17.0	83.0
Follow **very low calorie** diet (<1200 kcal/day)	72.9	27.1
Follow commercial /advertised diet	96.2	3.8
Exercise /sport	6.4	93.6
Do more exercise in everyday life (e.g. walking, gardening)	4.4	95.6
Leaflets on healthy behavior	41.5	58.5
Food diary	40.3	59.7
Nutritional education	32.8	87.2

*Diagnostic methods* based only on *inspection* were accepted less frequently by physicians qualified in family medicine only, than by doctors having two board examinations (16.1% vs 24.8%, p = 0.049).

The diagnosis of obesity based only on *body weight* measurement was made more frequently by doctors who treated only adults, than by family pediatricians (49.3 vs 32.8, p = 0.045).

*BMI-based diagnosis* was higher in cities (84.7%). It was only 71.2% in the capital and 74.3% in villages (p = 0.002).

*Waist circumference* was considered more frequently in pediatric than adult practices (79.1% vs 63.3%, p = 0.046). It was preferred by doctors qualified in family medicine only compared to those who had acquired more board qualifications (71.8% vs 61.7%, p = 0.035). Physicians working in the capital relied on waist circumference measurements rarely in comparison with doctors in other settings (57.0% vs 66.1%, p = 0.038).

*Waist/hip ratio* was calculated in 49.8% of adult and 64.2% of pediatric practices (p = 0.026). It was less commonly used in the capital than in other of settlements (43.6% vs 54.3%, p = 0.027). Older doctors realized its importance better than their younger colleagues did (56.2 ± 0.6 years vs 52.1 ± 0.7 years, p = 0.002). Doctors having board specialization / qualification in family medicine only regarded this approach more important than their colleagues with two qualifications (58.8% vs 50.5%, p = 0.038).

Differences were found in the mean age of doctors accepting *self-reported body weight* data from patients and those who insisted on weighing their patients (55.3 years vs 51.8 years, p = 0.002). This practice was significantly higher in the capital (p = 0.023).

Body weight measurements in underwear instead of outdoor clothing was preferred by older (55.6 years vs 53.7 years, p = 0.03) and underweight physicians and but fewer obese doctors followed suit (68% vs 37.5%, p = 0.04). The number of board examinations, being a resident, location and number of enrolled patients had no influence on this difference in the applied methodology.

Strict reduction in fat intake was emphasized by 86.0% of male and 92.9% of female physicians (p = 0.020) when giving nutritional advice. Eating (having a snack) between two main meals was prohibited more frequently by doctors with more qualifications (83.3% vs 74.9%, p = 0.044). They also recommended *personalized low-calorie diet* more frequently (83.3% vs 74.9%, p = 0.044). The doctors’ mean age preferring this type of diet was higher than that of their colleagues who did not (57.5 ± 0.7 years vs 53.2 ± 0.6 years, p = 0.014). Age was a significant contributor for agreeing to avoid some energy-dense dishes (55.2 ± 0.5 years vs 52.3 ± 0.9 years, p = 0.004). *Dietary advice* was provided less frequently in the capital than in other settlements (57.3% vs 67.8, p = 0.026) and also less frequently by more qualified doctors (63.4% vs 74.3%, p = 0.015). The mean age of doctors realizing the importance of dietary counseling was higher than that of those who did not (55.3 ± 0.6 years vs 52.7 ± 0.9 years, p = 0.010).

Doctors provided very different data about the achieved weight reduction of their obese patients following dietary counseling. No relation was found between the characteristic of practitioners and reported initial decrease in body weight, expressed in percent of the baseline weight. These date served a basis for *qualitative factorial (cluster)* analysis.

The theory and suspected reasons of developing obesity were discussed with the patients more frequently by female care providers than male physicians (64.6% vs 44.1%, p = 0.018). Fifty per cent of the residents also discussed this topic.

Ninety-three percent of the GPs recommended weight reducing programmes routinely. This figure was lower (83.0%) among family pediatricians. Doctors having a higher number of enrolled patients (above 1.600) provided dietary advice in a lower ratio (p = 0.06). Phone interview with patients, to monitor their achievement in weight reduction was used by 22.8% of GPs.

*Personalized physical activity programmes* were recommended by only 32.6% of the physicians. Female doctors consulted significantly longer with their obese patients and consultations were significantly shorter in practices with a higher number of enrolled patients (means: 12.1 vs 9.6 minutes). These figures were 10 vs 6 minutes by residents, respectively.

The vast majority of the doctors (96.3%) let their patients know about the expected changes in body weight. The recommended change in body weight within 6 months was 9.3 ± 6.6 percent of baseline body weight.

Following a diet recommended and advertised in the media was supported more frequently by male physicians (6.8% vs 2.5 %, p = 0.038), while their female colleagues quite often asked their patients to keep a record of the food they had consumed (64.8% vs 51.9%, p = 0.009).

### Outcomes of the factorial (cluster) analysis

Based on the characteristics of interest, the GPs were divided into three groups as follows:

*Cluster 1.* The smallest group (3%), consisted mainly of male physicians, usually with adult population in the capital. They spent the longest time in the practice (mean: 22 years) and had the shortest consultation time with their obese patients. They are less effective (3.5% of initial body weight) in the reported weight decrease of their patients. They have the lowest rating in vocation, preparedness and consultations.

*Cluster 2.* About one third of the doctors, mainly men, with the highest ratio of pediatric and mixed patient population. They spent less time in practice (mean: 20.5 years), and could be characterized with average length of consultation time, in vocation, preparedness and length of consultation. They were less effective in weight reduction (13.7%) and got lower rating in providing their patients with advice.

*Cluster 3*. It is the largest group, covering two thirds of the population. The doctors are mainly women with adult practices and of the typical geographical distribution. These doctors have spent the shortest time in practice (mean: 17.3 years). The length of consultations exceeds the average a bit. As far as achievement is regarded, practices in this cluster are the best (15%). The physicians’ vocation and preparedness are higher than average and they are also the best in counseling.

## Discussion

### Key results

The responders’ knowledge was different in some domains. There were many inconsistent findings and only few significant relations. Beside previous practice and recent knowledge, the physicians’ own lifestyle and BMI had a high impact on their attitudes and influenced their actual practice more than working location.

### Comparison with previous research

Different types of papers, qualitative and quantitative were also found, but, unfortunately most of them were incomparable. All the previous studies represented smaller, geographically closer settings even from large or continent-wide countries [13,15,16].

Despite increasing knowledge on obesity-related pathologies there were only small changes in the daily practice of doctors. Personal attitudes toward obesity have a great impact on professional practice of doctors. In countries with more advanced level of primary care other health professionals are also involved in the management of obesity. Health visitors, qualified nurses can contribute better in the prevention providing their patients with nutritional and life style advice [17]. In Hungary, only one practice nurse is employed by the GP.

Primary health care providers in some reported countries like Canada, the UK and the USA are not adequately equipped to deal with the *pediatric* obesity epidemic as yet [18-20].

In Hungary, multidisciplinary guidelines do not specifically address family physicians; adherence is low in general and GPs are not motivated financially. There is a need for a practical primary care guideline. Guidelines consider obesity as a complication rather than an entity. The latest guideline on obesity is not available for all GPs and their knowledge, practice and personal attitudes are quite different [21]. In the former undergraduate medical curriculum in Hungary, attended mainly by the “*older*” generation of GPs, obesity was not described as being a pathologic condition. Substantial proportion of practicing GPs worked previously in hospitals, acquired a board specification, mainly internal medicine and thereafter applied for a job in primary care. The daily practice of this generation of doctors is based mainly on personal experience and rarely on guidelines. It could be a reason why older doctors with higher own body weight/BMI are less active in the obesity management. Younger GPs usually participated in a residency program of family medicine. Moreover, guidelines do not have so great impact on professional practices of physicians in Hungary as they do in other countries.

Large studies has identified provider system and patient barriers to obesity care. Lack of obesity training during medical school and residency has been associated with significantly lower rates of discussing diet and exercise with obese patients [22-24].

The current practice of weight management and the attitudes and possible barriers towards treatment of overweight and obesity are not uniform [3]. Age, the physician’s gender, the physician’s weight, practice location, and current training status are all associated with some aspects of the physician’s attitudes and treatment practices [25].

Syntheses of the findings from the selected studies suggest that doctors and nurses of normal weight are more likely to use strategies to prevent obesity and give general advice to achieve weight loss than those who are overweight. The doctors’ and nurses’ own weight status was not closely related to their referral and assessment of overweight or obese patients. Associations with their relevant knowledge/skills and specific treatment behaviors were inconsistent and at the same time, patients’ lack of motivation was mentioned as a main barrier to successful treatment [4,7,9,11,16,23].

### Strengths and limitations of the study

Our evaluation was based on the nation-wide samples of Hungarian family physicians with high representation of different residency (from the capital to the small villages) and age cohort of GPs. Almost 10 % of Hungarian GPs in practice were questioned so their answer could be considered as representative. Distribution of the practice population of the questioned doctors was similar to the estimated Hungarian prevalence of obesity [1,2,26-28].

One of the *limitations* is that only the knowledge and attitudes of the residents could be evaluated, as they have a limited individual practice only.

Self-reported data do not always reflect the daily clinical practice and attitudes are often influenced by the daily workload in practice and the doctor’s actual mood. In order to achieve the higher participation rate, only cross sectional study were conducted. Unexpected finding, that GPs having more qualification had often lower performance was not evaluated.

## Conclusion

Effective assessment tools and treatment resources, dissemination of clinical practice guidelines, enhanced undergraduate medical education and postgraduate continuing medical education, and system-level changes are urgently needed to address this health problem.

Despite the growing worldwide epidemic of obesity, weight management is not adequately addressed in primary care, even not in Hungary.

New strategies should be developed to break down the GP’s barriers to weight management and to stimulate changes in GP’s attitudes.

More education of primary care physicians, specific examination techniques, and availability of community resources for obese persons is needed. Further research is needed to determine if interventions to increase the physicians’ knowledge will lead to less negative attitudes toward weight loss and extremely obese patients.

## Competing interests

The authors declare that they have no competing interests.

## Authors’ contributions

IR and PT planned the study; they collected the most relevant literature, organized the expert group and text writing, including final editing. Together with them, II, EH, LN and LK were the expert group members using their own literature search and they contributed in text writing as well. Dissemination and corresponding of the questionnaire was performed by IG, LRK, LM, CsM and JR with data collection and local/national literature and data search. All the data analysis and statistics was performed by ESz. All authors read and approved the final manuscript.

## Pre-publication history

The pre-publication history for this paper can be accessed here:

http://www.biomedcentral.com/1471-2296/14/156/prepub
